# A community-based screening programme to detect non-alcoholic fatty liver using FibroScan among healthy Indian adults

**DOI:** 10.6026/973206300223332

**Published:** 2026-06-30

**Authors:** Singhai Abhishek, Joshi Rajnish, Yadav Vikas, Ingle Vaibhav, Manoria Piyush

**Affiliations:** 1 Department of Medicine, AIIMS, Bhopal, Madhya Pradesh, India; 2 Department of Environmental Health and Epidemiology, ICMR- National Institute for Research in Environmental Health, Bhauri, Bhopal, Madhya Pradesh, India; 3 Private Practitioner, Consultant Gastroenterologist, Manoria Hospital & Research Centre, Bhopal, Madhya Pradesh, India

**Keywords:** NAFLD, BMI, LDL, public health, obesity

## Abstract

Non-alcoholic fatty liver disease (NAFLD) remains substantially underdiagnosed in the general Indian population despite its rapidly increasing burden and close association with metabolic disorders. Therefore, it is of interest to evaluate the prevalence of NAFLD among Indian adults. Hence, 490 participants were included in the study. We identified NAFLD in 44.5% of population. NAFLD now poses a significant health challenge in India. Its strong association with obesity, diabetes, and dyslipidemia highlights the urgent need for targeted public health strategies and early intervention.

## Background:

NAFLD has emerged as a major global liver health issue, with estimates suggesting it affects up to 30% of adults worldwide [1]. NAFLD encompasses a spectrum that includes simple fat accumulation (steatosis), steatohepatitis (NASH), fibrosis, and in severe cases, cirrhosis [2, 3]. Nonalcoholic fatty liver disease (NAFLD) has recently been reclassified as metabolic dysfunction-associated steatites liver disease (MASLD) to better reflect its underlying metabolic etiology rather than the exclusion of alcohol consumption; however, as this nomenclature change occurred after the initiation of our study, the term NAFLD has been retained throughout this article for consistency [4]. Numerous Indian studies have identified NAFLD rates between 20% and 50% in urban populations [5]. However, there remains a notable absence of community-based data from central India, particularly involving the use of advanced diagnostic modalities [6]. Prior research has commonly relied on ultrasonography, which, while widely accessible, lacks the sensitivity to detect mild steatosis and does not provide information about hepatic fibrosis [7]. Traditional noninvasive scores such as FIB-4 and NAFLD fibrosis score have shown limited reliability in population screening, highlighting the need for more accurate tools [8]. FibroScan (vibration-controlled transient elastography) has emerged as a cornerstone noninvasive modality for assessing liver steatosis and fibrosis, with high diagnostic accuracy and applicability in large-scale screening [9]. The controlled attenuation parameter (CAP) obtained via FibroScan allows quantitative assessment of hepatic steatosis, correlating well with metabolic risk factors [10]. Recent studies have reported a substantial prevalence of NAFLD and varying degrees of fibrosis in screened populations, underscoring the importance of early identification [11, 12]. Therefore, it is of interest to screen asymptomatic individuals from Bhopal's urban regions to explore how feasible it would be to adopt community-wide liver screening using a non-invasive approach like using FibroScan technology to enable earlier identification and management of liver disease.

## Materials and Methods:

This community-based, cross-sectional study was undertaken between March 2022 and January 2025 across ten urban regions of Bhopal. These regions were selected randomly by computer generated numbers among 52 wards of Bhopal. The target group included adults aged 18 years or older. Eligibility required voluntary participation through written informed consent. Individuals with significant alcohol use, existing diagnosis of cirrhosis, prior detection of hepatitis B or C, known biliary pathology, or current pregnancy were excluded to eliminate confounding factors affecting liver health. This Sample size (n) calculated using the following formula: n = N*X / (X + N - 1), where, X = Z2α/2 *p*(1-p) / MOE2, and Zα/2 is the critical value of the normal distribution at α/2 (e.g. for a confidence level of 95%, α is 0.05 and the critical value is 1.96), MOE is the margin of error (5%), p is the sample proportion (30% in previous studies [13] and N is the population size. Note that a Finite Population Correction has been applied to the sample size formula. Calculated sample size was 323. This means 323 or more measurements are needed to have a confidence level of 95% that the real value is within ±5% of the measured value. Considering 1.5 design effect for cluster sampling and 5% nonresponse rate, final sample size was 500. Outreach activities were led by trained personnel, including a nurse and a research assistant, who provided health education and invited residents to undergo non-invasive liver assessments using FibroScan technology. The procedural overview of the study is outlined in Figure 1 (see PDF). Participants underwent a set of physical assessments performed by research assistants, which included measuring body weight, height, waist and hip circumferences. These values were used to derive body mass index (BMI) and waist-to-hip ratio. Blood pressure was measured by a qualified nurse under standard resting conditions. After a 12-hour fast, participants were scheduled for abdominal ultrasonography alongside blood testing. The biochemical investigations included fasting plasma glucose (FPG), lipid profile, alanine aminotransferase (ALT/SGPT), hepatitis B surface antigen (HBsAg) or anti-hepatitis C virus (HCV) antibodies testing to screen for metabolic and infectious contributors to liver pathology. All participants underwent liver stiffness assessment using FibroScan, a non-invasive imaging modality based on transient elastography. To ensure measurement validity, each scan consisted of at least 10 acceptable readings, with a requirement that 60% or more be successful and that the interquartile range remain within 30% of the median value. Results were recorded in kilopascals (kPa), reflecting tissue stiffness. Additionally, FibroScan provided a Controlled Attenuation Parameter (CAP) score, an estimate of hepatic fat accumulation, measured in decibels per meter (dB/m). CAP values typically fall between 100 and 400 dB/m and correspond to specific grades of hepatic steatosis. When the CAP value falls between 238 and 259 dB/m, it indicates Grade 1 steatosis, which is considered mild, with approximately 11% to 33% fat involvement in the liver. A CAP measurement between 260 and 289 dB/m corresponds to Grade 2 steatosis, classified as moderate, with an estimated 34% to 66% fat content. When the CAP value is 290 dB/m or higher, it reflects Grade 3 steatosis, which is severe, with fat involvement exceeding 67%. Liver fibrosis involves the formation of scar tissue as a result of chronic liver injury. The severity of fibrosis can be assessed non-invasively using Liver Stiffness Measurement (LSM) provided by the FibroScan. In individuals without liver pathology, LSM values generally fall between 2 and 6 kilopascals (kPa). While the device is capable of measuring stiffness up to 75 kPa, such elevated readings are rarely encountered in clinical practice. This study was approved by institutional ethical committee.

## Results and Discussion:

The recruitment process for this study began with the identification of 654 asymptomatic adult individuals from the community. Out of these, 90 declined to enroll. Another 35 participants were excluded on account of significant alcohol consumption, and an additional 4 women were excluded due to pregnancy. This left 525 eligible participants who underwent liver assessment via FibroScan. During the subsequent evaluation phase, 15 participants opted out of the blood sampling, 10 declined the abdominal ultrasound and 10 were excluded after testing positive for either HBsAg or HCV antibodies. As a result, comprehensive clinical and diagnostic data were obtained for 490 individuals, which formed the final dataset used for analysis. A visual representation of participant flow is provided in Figure 1 (see PDF). FibroScan assessment revealed that 44.5% of participants in the study exhibited evidence of NAFLD. Among those diagnosed, a range of steatosis severity was observed. Mild steatosis, corresponding to grade S1, was identified in 14.9% of participants. An additional 11.2% exhibited moderate fat accumulation in the liver, classified as grade S2, while 18.4% of individuals showed signs of advanced steatosis, or grade S3, reflecting a severe degree of hepatic fat infiltration. Liver fibrosis (F2-F3) was seen in only 11 (2.2%) participants. The distribution of steatosis grades and fibrosis grades are summarized in Table 1. Table 2 shows the sociodemographic and clinical profile of the study participants. The mean age of the study cohort was 36.3 years (±10.4), indicating a predominantly young adult population, with 81.4% of individuals aged below 50 years. A higher proportion of participants were male, comprising 63.1% of the total sample. Anthropometric evaluation revealed a mean BMI of 26.7 kg/m^2^ (±4.2), placing the average participant in the overweight category. The average waist circumference was 95.8 cm (±10.3), while the mean hip circumference measured 101.8 cm (±8.8). Consequently, the average waist-to-hip ratio was found to be 0.94 (±0.05), suggesting a trend toward central obesity among participants. In terms of liver fat quantification, the mean CAP score among the screened adults was 240.1 dB/m (±53.1), indicating varying degrees of hepatic steatosis. Biochemical markers showed mean fasting plasma glucose (FPG) of 99.7 mg/dL (±29.7) and mean SGPT (ALT) level of 39.5 U/L (±43.7). The average total cholesterol level was 169.1 mg/dL (±41.8), while mean serum triglyceride was 146.6 mg/dL (±99.9). Among co-morbidities, 6 participants reported having hypertension, 13 had been diagnosed with diabetes mellitus, and 2 individuals had known hypothyroidism. Table 3 displays the results of a logistic regression analysis assessing the impact of clinical and demographic parameters on the development of NAFLD. To assess the influence of individual risk factors on NAFLD, univariate and multivariable logistic regression analyses were employed to estimate both unadjusted and adjusted associations. Bivariate analysis showed significant associations between NAFLD and several variables, including older age, male sex, elevated BMI, higher waist-to-hip ratio, increased FPG, and elevated levels of triglycerides, total cholesterol, LDL, and VLDL. In the multivariate model, after controlling for confounders, age, male sex, BMI, FPG, and LDL levels remained independently and significantly associated with NAFLD, as detailed in Table 3.

According to our study, the sensitivity, specificity, positive predictive value, and negative predictive value of ultrasonography for diagnosing NAFLD are 62.4%, 80.9%, 72.3%, and 72.8%, respectively, when compared to the gold standard FibroScan (Table 4). This community-based screening initiative, employing FibroScan technology, uncovered a notable prevalence of NAFLD among asymptomatic adults, encompassing a spectrum of steatosis severity. As one of the earliest studies in Central India to apply FibroScan for non-invasive NAFLD detection, it offers region-specific epidemiological insights and highlights the critical need for early diagnosis strategies in comparable urban populations. The results align with urban prevalence estimates of NAFLD, such as the 44.6% figure reported by Shalimar *et al.* in a large-scale meta-analysis incorporating 62 datasets from 15 studies across India, which yielded a pooled prevalence of 38.6% [5]. In contrast, a lower prevalence of 32% was observed by Mohan *et al.* using ultrasound among urban adults in South India, illustrating the shortcomings of ultrasonography in identifying early or mild steatosis [6]. The application of FibroScan in our study allowed for more precise, quantitative assessment of both hepatic fat and fibrosis, thereby improving the reliability of screening outcomes in the community setting. When compared to international data, our observed prevalence surpasses those reported by Riazi *et al.* (32.4%) and Li *et al.* (33.5%) [7, 14]. Indicating that India, and particularly its central region, may be becoming a hotspot for NAFLD. This trend reflects a broader global shift in metabolic liver disease patterns, with increasing burdens now seen in Asian populations that were historically less affected. The elevated prevalence in our study can likely be explained by urban lifestyle factors. For instance, Asadullah *et al.* found that NAFLD was present in 55.1% of urban dwellers compared to just 26.2% in rural populations in North India - a difference attributed to higher levels of obesity, insulin resistance, and physical inactivity in urban settings [15]. Our study population exhibits comparable demographic and metabolic characteristics. Evidence from hospital-based studies further illustrates the strong association between NAFLD and metabolic disorders. Agarwal *et al.* reported 57.2% prevalence in patients with type 2 diabetes, highlighting significant correlations with hypertension, central obesity, dyslipidemia, and cardiovascular risk [16]. Jayarama and Sudha similarly noted that NAFLD affected 60% of diabetics compared to just 20% of non-diabetics, with the condition strongly linked to elevated BMI and poor glycemic control [1 7]. Several other Indian studies have supported these associations [18, 19, 20]. Gupte *et al.* found that among asymptomatic type 2 diabetics, 87.5% of those diagnosed with fatty liver via ultrasound had biopsy-confirmed NASH, and over one-fifth had fibrosis-even in the absence of obesity or elevated liver enzymes [21]. These insights emphasize the limitations of traditional screening parameters like BMI and liver function tests and highlight the value of FibroScan in early diagnosis. FibroScan provided considerable diagnostic advantages over conventional ultrasound in our study. According to Macabuag-Oliva *et al.* it demonstrated greater sensitivity (100% vs. 82.5%) and specificity (86.5% vs. 32.4%) for identifying both hepatic steatosis and fibrosis, especially among individuals with metabolic syndrome and type 2 diabetes [13]. Its non-invasive nature, reproducibility, and quantitative output make it a suitable tool for widespread use in population-level screening programmes. Indian guidelines provide evidence-based recommendations for NAFLD screening, diagnosis, and treatment in people with type 2 diabetes. Important conclusions include the requirement that all people with type 2 diabetes undergo screening, with a focus on those who have high-risk characteristics including obesity and metabolic syndrome [22, 23]. Harshini *et al.* from Puducherry found that the prevalence of NAFLD among individuals at high risk was 22.8% (95% CI: 18.7%-27.5%), and it was greater among men (35.2%), those with diabetes mellitus (33.3%), and those who were obese (40.2%) [24]. some limitations of our study must be acknowledged. The findings are specific to selected urban localities within Bhopal and may not be broadly generalizable. Moreover, although FibroScan reliably detects steatosis and fibrosis, it cannot distinguish between simple fatty liver and NASH. Participation may also have been biased towards individuals more conscious of their health. Nevertheless, the study's standardized procedures, substantial community engagement, and non-invasive approach underscore the feasibility and value of conducting liver health assessments at the community level. Further longitudinal research is necessary to monitor the natural history of subclinical NAFLD cases identified through such screenings and to explore the cost-effectiveness of integrating FibroScan into broader public health frameworks. In summary, this study exposes a considerable unrecognized burden of NAFLD in urban Indian populations and advocates for the inclusion of non-invasive liver screening in strategies aimed at combating metabolic disorders. This study advances current knowledge by providing one of the first large community-based FibroScan screening datasets for NAFLD from Central India, particularly among asymptomatic urban adults. Unlike earlier Indian studies [25, 26] that primarily relied on ultrasonography, this study utilized vibration-controlled transient elastography with CAP measurement, enabling more accurate detection of both hepatic steatosis and fibrosis. The findings demonstrate a high burden of previously undiagnosed NAFLD (44.5%) in apparently healthy individuals and reinforce the strong association of NAFLD with obesity, dysglycemia, and dyslipidemia. The study also highlights the limitations of conventional ultrasonography in population screening and supports the feasibility of incorporating non-invasive FibroScan-based liver assessment into community screening programmes for early identification of metabolic liver disease in India. These findings contribute important region-specific epidemiological data to the evolving literature on NAFLD in South Asian populations.

## Conclusion:

We report high prevalence of NAFLD among Indian adults. Continued research, particularly longitudinal studies, will be critical to understanding the natural course of NAFLD. This shows the long-term impact of community-based screening and prevention strategies.

## Figures and Tables

**Table 1:** NAFLD grades in all study subjects

		**Frequency**	**Percent**	**Valid Percent**	**Cumulative Percent**
NAFLD (Steatosis	NAD	272	55.5	55.5	55.5
grades)	S1	73	14.9	14.9	70.4
	S2	55	11.2	11.2	81.6
	S3	90	18.4	18.4	100
	Total	490	100	100	
NAFLD (Fibrosis grades)	F0-F1	479	97.8	97.8	97.8
	F2	8	1.7	1.7	1.7
	F3	3	0.5	0.5	0.5
	F4	0	0	0	0
	Total	490	100	100	100
NAD= No abnormality detected

**Table 2:** Table showing the sociodemographic characteristics of the study participants

**Group statistics Variables**		**N**	**Mean**	**Std. Deviation**	**Std. Error Mean**	**t-test for Equality of Means**	**df**	**Sig. (2-tailed)**	**Mean Difference**	**Std. Error Difference**	**Lower CI (95% Confidence Interval of the Difference)**	**Upper CI (95% Confidence Interval of the Difference)**
Waist-Hip Ratio	Controls	272	0.9	0.06	0.01	-5.56	488	0	-0.03	0.005	-0.04	-0.02
	Cases	218	0.94	0.05	0.01							
Age	Controls	272	31.95	8.95	0.54	-4.99	488	0	-4.37	0.87	-6.09	-2.64
	Cases	218	36.33	10.42	0.7							
Height	Controls	272	162.76	9.12	0.55	-3.17	488	0.002	-2.63	0.82	-4.25	-1.01
	Cases	218	165.39	9.08	0.61							
Weight	Controls	272	62.2	11.77	0.71	-9.58	488	0	-11.05	1.15	-13.31	-8.78
	Cases	218	73.25	13.73	0.93							
BMI	Controls	272	23.44	3.91	0.23	-8.9	488	0	-3.28	0.36	-4.01	-2.55
	Cases	218	26.73	4.23	0.28							
Waist Circumference	Controls	272	86.12	9.66	0.58	-10.66	488	0	-9.63	0.9	-11.4	-7.85
	Cases	218	95.75	10.25	0.69							
Hip Circumference	Controls	272	95.11	10.2	0.61	-7.67	488	0	-6.7	0.87	-8.42	-4.99
	Cases	218	101.82	8.81	0.59							
Fasting blood sugar	Controls	272	90.36	16.77	1.01	-4.39	488	0	-9.36	2.13	-13.54	-5.17
	Cases	218	99.72	29.72	2.013							
SGPT	Controls	272	25.77	17.63	1.069	-4.74	488	0	-13.76	2.9	-19.47	-8.05
	Cases	218	39.54	43.66	2.957							
Cholesterol	Controls	272	156.65	34.84	2.112	-3.59	488	0	-12.43	3.46	-19.23	-5.63
	Cases	218	169.11	41.76	2.828							
Triglyceride	Controls	272	109.48	58.47	3.545	-5.13	488	0	-37.15	7.24	-51.37	-22.92
	Cases	218	146.64	99.98	6.771							
HDL	Controls	272	41.54	12.11	0.734	-0.01	488	0.98	-0.021	1.23	-2.451	2.4
	Cases	218	41.56	15.26	1.033							
LDL	Controls	272	91.95	29.33	1.778	-3.21	488	0.001	-9.24	2.88	-14.9	-3.58
	Cases	218	101.2	34.4	2.329							
VLDL	Controls	272	22.14	12.93	0.784	-4.77	488	0	-7.37	1.54	-10.4	-4.34
	Cases	218	29.52	20.95	1.418							
*Subjects with CAP score ≥238 dB/m are cases while subject with CAP score <238 dB/m are controls.

**Table 3:** Logistic regression analysis of effect of demographic and clinical parameter on development of NAFLD

**Sr. No.**		**Bivariate analysis**		Multivariate analysis (Adjusted OR)*	
	Parameters	OR (lower CI - upper CI)	P value on bivariate analysis	OR (lower CI - upper CI)	P value on multivariuate analysis
1	Age	1.048	<0.001	1.035	0.002
2	Sex(1)@	0.551	0.002	0.477	0.002
3	Height	1.032	0.002	-	-
4	Weight	1.075	<0.001	-	-
5	BMI	1.23	<0.001	1.211	<0.001
6	Waist Circumference	1.11	<0.001	-	-
7	Hip Circumference	1.094	<0.001	-	-
8	Waist-Hip Ratio	5303.033	<0.001	18.522	0.089
9	Alcohol(1)#	2.356	0.097	1.872	0.264
10	Fasting blood sugar	1.026	<0.001	1.016	0.006
11	SGPT	1.025	<0.001	1.021	<0.001
12	Cholesterol	1.009	<0.001	0.98	0.078
13	Triglyceride	1.007	<0.001	1.006	0.173
14	HDL	1	0.986	1.004	0.752
15	LDL	1.009	0.002	1.028	0.018
16	VLDL	1.031	<0.001	1.011	0.632
*Multivariate analysis- Model 1 was run on age,
male sex, BMI, waist to hip ratio and insignificant alcohol consumption.
Model 2 was run on fasting blood sugar, SGPT, serum cholesterol, serum triglycerides, HDL, LDL and VLDL.

**Table 4:** NAFLD diagnosis by USG and FibroScan

**NAFLD**		**FibroScan diagnosis (Gold standard)**		**Total**
		Case	NAD	
USG diagnosis	Case	136	52	188
	NAD	82	220	302
Total		218	272	490
USG= ultrasound abdomen
